# Transcriptomic Analysis Reveals Novel Mechanisms Underlying Neutrophil Activation Induced by High Salt

**DOI:** 10.3390/ijms27021083

**Published:** 2026-01-21

**Authors:** Ignacio Mazzitelli, Lucía Bleichmar, Federico Rivelli, Ingrid Feijoo, Alan Adamczyk, Gonzalo Cabrerizo, Fernando Erra Díaz, Jorge Geffner

**Affiliations:** Facultad de Medicina, Instituto de Investigaciones Biomédicas en Retrovirus y SIDA (INBIRS), Universidad de Buenos Aires, CONICET, Buenos Aires C1121ABG, Argentina; mazzitelli.ignacio@gmail.com (I.M.); lucia.bleich@gmail.com (L.B.); fmrivelli@gmail.com (F.R.); ingridsoledadfeijoo@hotmail.com (I.F.); alanadamczyk@live.com (A.A.); gonzalocabrerizo11@gmail.com (G.C.); fererrad@gmail.com (F.E.D.)

**Keywords:** neutrophils, sodium chloride, inflammation, mitochondrial ROS

## Abstract

Elevated sodium concentrations are commonly observed in tumors and sites of inflammation. Previous studies have shown that high salt levels modulate the phenotype and function of CD4^+^ and CD8^+^ T cells, regulatory T cells, and macrophages. In this study, we performed transcriptomic studies that revealed profound alterations in the neutrophil transcriptome upon high salt exposure, with changes that significantly exceeded those triggered by conventional agonists. By integrating transcriptomic data with functional assays, our findings suggest that high salt-induced neutrophil activation involves mitochondrial ROS production, which subsequently activates p38 MAPK and engages FOS-, Bruton’s tyrosine kinase (BTK)-, and cyclooxygenase 2 (COX2)-dependent pathways. Remarkably, the plasticity of the neutrophil transcriptome in response to high salt was further evidenced by the upregulation of genes typically associated with other cell types, including semenogelin 1 (*SEMG1*), intercellular adhesion molecule-4 (*ICAM4*), tripartite motif69 (*TRIM69*), amphiregulin (*AREG*), oncostatin (*OSM*), and transducer of ERBB2-1 (*TOB1*), suggesting a broader role for neutrophils in different biological processes beyond their participation in innate immunity.

## 1. Introduction

Neutrophils play a critical role in the immune response against bacteria and fungi [1]. However, the traditional perspective of neutrophils as terminally differentiated, short-lived cells with a role restricted to antimicrobial immunity and host damage has changed over the past two decades. By releasing a wide range of biological mediators, neutrophils have been shown to play a central role in the mechanisms leading to tissue repair, as well as in modulating the function of different immune cells, thereby influencing both the resolution and persistence of inflammatory processes [2,3,4,5,6]. Moreover, neutrophils are also able to suppress anti-tumor immunity, and consistent with this, high frequencies of intratumoral neutrophils are often associated with a poor outcome in cancer [7,8,9,10,11].

The functional versatility of neutrophils can be explained by single-cell RNA sequencing studies showing the existence of different neutrophil subsets under both physiological and pathologic conditions [8,12,13]. On the other hand, and contrasting with the traditional view showing neutrophils as poorly plastic cells, studies carried out over the last 20 years have demonstrated not only that neutrophils comprise distinct subsets with different phenotypic and functional properties but also that they are able to display rapid and broad transcriptional firing upon stimulation with cytokines, pathogen-associated molecular patterns (PAMPs), and damage-associated molecular patterns (DAMPs) [14,15,16]. Analysis of differentially expressed genes (DEGs) in normal-density neutrophils from individuals treated with G-CSF has shown the induction of a profound transcriptome reprogramming markedly higher than that observed in monocytes, highlighting the remarkable plasticity that neutrophils show in vivo [4]. Marked changes in the neutrophil transcriptome have also been described in the course of cancer [17,18,19], tuberculosis [20,21], influenza [22], and SARS-CoV-2 infection [23]. As expected, in vitro activation of neutrophils also results in broad changes in the transcriptome. Exposure of human neutrophils to LPS, opsonized bacteria, chemotactic peptides, and IL-8 has been shown to induce a set of alterations in gene expression which includes many transcription factors, cytokines, chemokines, and pattern recognition receptors (RRPs) [24,25,26]. It should be emphasized that, although the average lifespan of circulating neutrophils is less than 18–24 h, their infiltration into inflammatory foci or tumors has been shown to extend their half-life to 4–7 days, allowing for the acquisition of phenotypic and functional changes [2,27,28].

The phenotype and function of neutrophils in challenged tissues can be modulated by pathogen-associated molecular patterns (PAMPs), damage-associated molecular patterns (DAMPs), cytokines, and a variety of biological mediators [29,30]. Relatively little attention has been given to the immunomodulatory effects triggered by alterations in the physicochemical properties of the microenvironments where neutrophils operate, particularly the increased sodium concentration in peripheral tissues. High concentrations of sodium can be found in the skin as a consequence of high salt diets [31]. Moreover, they are commonly found at inflammatory foci [32,33,34], as well as in lymphoid organs [35] and tumors [36,37,38]. High salt intake is now endemic worldwide [39]. It has been clearly associated with the induction of a chronic low-grade inflammation promoting hypertension [40], cardiovascular and kidney damage [41,42], and a more severe course of different inflammatory diseases [43,44,45]. This association can be explained by the ability of high salt (HS) to modulate the function of immune cells [46]. Previous studies have shown that HS promotes the differentiation of the CD4^+^ T cells into a TH17 profile [43,47], impairs regulatory T cell function [48], improves CD8^+^ T cell function in the tumor microenvironment [49], and favors the acquisition of a proinflammatory M1 phenotype by macrophages [50,51].

We have previously reported that HS induces a paradoxical effect on human neutrophils: a suppression of neutrophil function at early times followed by a marked activation of a number of neutrophil responses at later times, including degranulation, production of reactive oxygen species (ROS), and release of IL-8 and TNF-α [52]. In order to obtain a comprehensive overview of the changes induced by sodium exposure, we analyzed here its impact on the neutrophil transcriptome. Our observations indicate that exposure to high Na^+^ concentrations induces dramatic changes in the neutrophil transcriptome. These changes not only help explain the mechanisms underlying the acquisition of an inflammatory signature by HS-exposed neutrophils but also broaden the range of functions potentially mediated by neutrophils.

## 2. Results

### 2.1. Analysis of Neutrophil Transcriptional Changes Induced by High Salt

**Figure 1:**
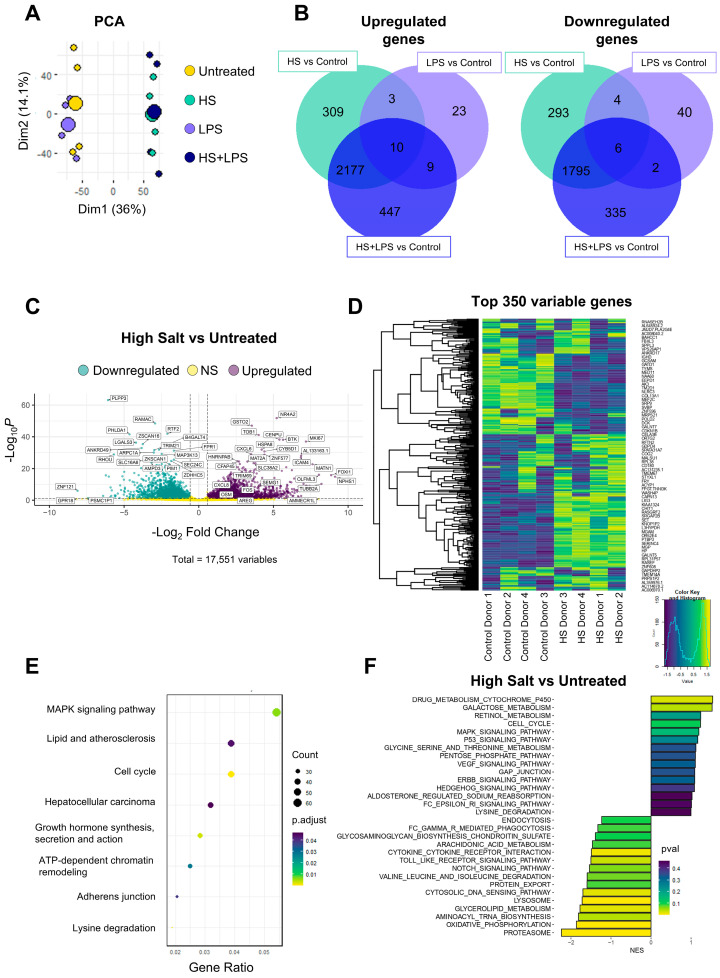
Transcriptional landscape of neutrophils exposed to high salt. (**A**) RNA-seq of human neutrophils cultured for 4 h in the presence or absence of HS (NaCl 50 mM) with or without LPS (100 ng/mL). Principal Component Analysis (PCA) of the normalized data z-scores are shown (*n* = 4 for each condition). (**B**) Venn diagrams illustrating the intersections of upregulated and downregulated DEGs among neutrophils exposed to HS, LPS, and HS plus LPS compared with untreated neutrophils. (**C**) Volcano plots show the results of differential gene expression analysis between HS-exposed neutrophils compared with untreated neutrophils. Differentially expressed genes (DEGs) are defined as FC > 0,58, adjusted *p*-value < 0.05. (**D**) Top 350 highly variable genes of donor normalized z values are shown. (**E**) Over-representation analysis (ORA) of upregulated genes when comparing HS-exposed neutrophils with untreated neutrophils. Dot size reflects the number of genes associated with each KEGG pathway, while the color scale indicates the adjusted *p*-values. (**F**) Gene Set Enrichment Analysis (GSEA) was performed to identify enriched pathways when comparing HS-exposed neutrophils with untreated neutrophils. The fold changes from differential expression analysis were used to assess pathway enrichment using curated KEGG metabolic signaling pathways. Pathways with positive or negative enrichment scores (ESs) were separately analyzed and the top 15 upregulated and downregulated pathways were visualized in a bar plot. Pathways were sorted based on normalized enrichment scores (NESs), and the color scale represents the adjusted *p*-values for each pathway.

**Figure 2:**
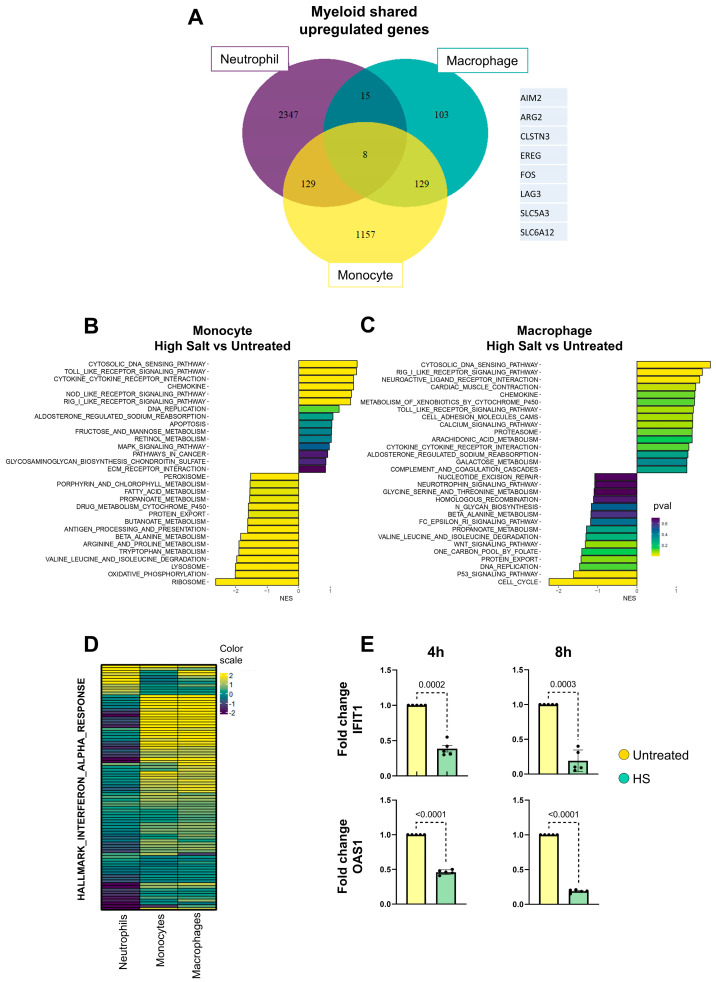
Comparison of transcriptome changes induced in neutrophils, monocytes, and macrophages by exposure to high salt. (**A**) Venn diagrams illustrating the limited overlap of upregulated DEGs of HS-exposed neutrophils, monocytes, and macrophages when compared with untreated cells. The list of the 12 genes showing an increased expression in the three cell types is shown. (**B**,**C**) Gene Set Enrichment Analysis (GSEA) of monocytes (**B**) and macrophages (**C**) was performed to identify enriched pathways when comparing HS-exposed cells with untreated cells. The fold changes from differential expression analysis were used to assess pathway enrichment using curated KEGG metabolic signaling pathways. Pathways with positive or negative enrichment scores (ES) were separately analyzed and the top 15 upregulated and downregulated pathways were visualized in a bar plot. Pathways were sorted based on normalized enrichment scores (NESs), and the color scale represents the adjusted *p*-values for each pathway. (**D**) Heatmap showing the log2 fold changes in genes involved in the Interferon Alpha Response (from HALLMARK gene set) in neutrophils, monocytes, and macrophages exposed to HS compared with untreated cells. Only genes that were consistently present in all three cell types were included. The color scale represents the magnitude of log2 fold changes. Rows are clustered based on gene expression patterns, while columns represent the different cell types. (**E**) Neutrophils (10 × 10^6^/mL) were cultured for 4 h and 8 h at 37 °C in 5% CO_2_ in culture medium supplemented or not with 50 mM of NaCl. Cells were then lysed, RNA extracted and retrotranscribed, and the relative expression levels of *IFIT1* and *OAS1* were analyzed using qPCR, using *ACTB* as a housekeeping gene. Results represent the mean ± SEM of three different donors.

To gain insight into the atypical pattern of neutrophil activation induced by HS, we performed a transcriptome analysis of neutrophils from healthy donors cultured for 4 h in medium supplemented with HS and/or LPS. In light of previous studies reporting that tumor and inflammatory processes are associated with elevations in local sodium concentrations within the range of 30 to 100 mM [24,26,28,30], we performed our experiments in culture medium supplemented with 50 mM NaCl. Preliminary experiments showed, as expected, that neutrophil viability at 4 h of culture was higher than 95% for all the experimental conditions, evaluated by annexin V staining and flow cytometry (Supplementary Figure S1A). Moreover, as we previously described [52], either high salt- or LPS-treated neutrophils produced high levels of IL-8 when evaluated after 8 h of stimulation (Supplementary Figure S1B). RNA sequencing (RNA-seq) data were generated from neutrophils isolated from four healthy donors and gene expression can be interactively accessed at https://ignacio21.shinyapps.io/NeutrophilsUBA/ (accessed on 3 October 2024). Characteristics of these donors are described in Supplementary Table S1. Principal Component Analysis (PCA) performed after normalizing the data by donor (“z-score”) shows that exposure to high sodium represents the major source of transcriptional variation compared to untreated cells (Figure 1A). In fact, the analysis of DEGs revealed that, compared with untreated neutrophils, those exposed to HS or LPS showed 2499 and 45 upregulated transcripts, and 2098 and 52 downregulated transcripts, respectively, indicating that HS induces greater changes in the neutrophil transcriptome, compared with the changes induced by LPS. Supplementary Table S2 shows the list of DEGs comparing untreated vs. HS-treated neutrophils. As expected, simultaneous exposure to HS and LPS also resulted in profound changes in the transcriptome; the upregulation of 2643 transcripts and the downregulation of 2138 transcripts (Figure 1B). Most of the changes induced by HS plus LPS were also observed in neutrophils treated only with HS (Figure 1B), suggesting a dominant role for HS in the transcriptomic changes induced in neutrophils exposed simultaneously to HS plus LPS. We conclude that exposure to HS notoriously reprograms the genetic architecture of neutrophils. The volcano plot shown in Figure 1C illustrates the distribution of genes according to their Log2 (FC) and FDR (−Log10 adjusted *p*-value), while the analysis of the 350 genes showing the greatest variability clearly illustrated differences between untreated and HS-treated neutrophils (Figure 1D). To examine how HS modulated different biological processes, we performed an over-representation analysis (ORA) using the KEGG pathway database, focusing on the set of upregulated genes. As expected, an overrepresentation of genes involved in MAP kinase signaling, typically involved in cellular response to stressful conditions such as exposure to ultraviolet light, irradiation, heat shock, osmotic stress, and proinflammatory cytokines [53] was observed (Figure 1E). Gene Set Enrichment Analysis (GSEA), on the other hand, showed that genes associated with oxidative phosphorylation, proteasome, and cytosolic DNA sensing were markedly downregulated, while genes associated with drug metabolism via cytochrome P450, galactose and retinol metabolism, and MAPK activation were upregulated (Figure 1F). Interestingly, by re-analyzing published datasets from studies aiming to characterize the proinflammatory effects induced in vitro by HS on human monocytes [54] and macrophages [51], a very different picture was observed. In fact, only few transcripts were similarly upregulated in response to HS by neutrophils (our own data) compared with monocytes and macrophages (Figure 2A), thus suggesting that even in the myeloid compartment there are divergent transcriptomic responses induced by HS dependent on the cell type analyzed (Figure 2B,C). In fact, some genes seem to be regulated in an opposite way in neutrophils compared with monocytes and macrophages. For example, cytosolic DNA-sensing pathways and interferon-stimulated genes were markedly downregulated in neutrophils but they represent one of the most upregulated pathways in both monocytes and macrophages (Figure 2D). Considering the fact that the transcriptomic data from monocytes and macrophages were obtained after 72 h and 24 h of culture, respectively, while our data in neutrophils were obtained at 4 h of culture, we analyzed the RNA transcripts for interferon-induced protein with tetratricopeptide repeats 1 (*IFIT1*) and 2′-5′-oligoadenylate synthetase 1 (*OAS1*) at 8 h of culture to determine whether longer times of neutrophil culture in the presence of HS resulted in a higher expression of these two interferon-stimulated genes. Similar inhibition in the expression of *IFIT1* and *OAS1* was observed at either 4 h or 8 h of culture (Figure 2E).

### 2.2. High Salt Profoundly Impacts the Production of Chemokines by Neutrophils

**Figure 3:**
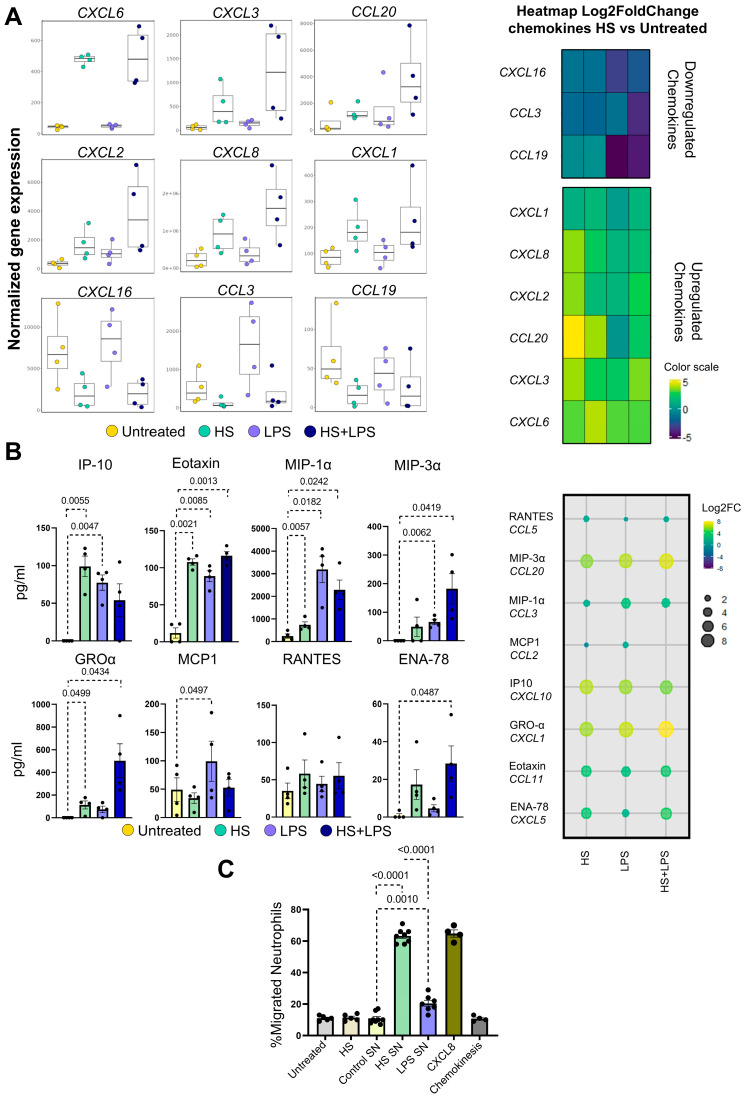
Impact of high salt on the production of chemokines by neutrophils. (**A**) Normalized gene expression values for chemokine genes (left panels) and heatmap displaying log_2_ fold changes (HS vs. untreated neutrophils) for differentially expressed chemokine genes (right panel). Genes with fewer than 250 counts across all samples were excluded from the analysis. (**B**) Neutrophils (2 × 10^6^/mL) were cultured for 18 h at 37 °C in culture medium supplemented, or not, with NaCl 50 mM and/or LPS (100 ng/mL). Chemokine levels were determined by CBA in culture supernatants. Results are shown as absolute values (left panels) and as log2 FC comparing HS-, LPS-, and HS plus LPS-treated neutrophils with untreated neutrophils (right panel). (**C**) Neutrophil chemotaxis assays were performed using a transwell chamber, untreated neutrophils, and supernatants (SNs) from untreated, HS-treated, and LPS-treated neutrophils harvested after 18 h of culture, as described in Materials and Methods. Results are expressed as the mean ± SEM of 4–8 donors.

We have previously shown that HS induces the production of IL-8 by neutrophils at levels as high as those induced by LPS [52]. Figure 3A reveals that exposure to HS not only stimulates the transcription of IL-8 but also the transcription of a wide range of chemokine genes involved not only in the mobilization of neutrophils (*CXCL6*, *CXCL3*, *CXCL2*, and *CXCL1*) but also in the recruitment of T, B, and dendritic cells (*CCL20*). By contrast, HS appears to inhibit the transcription of some chemokine genes such as *CXCL16*, *CCL3*, and *CCL19*. The ability of HS-exposed neutrophils to act as an important source of chemokines was confirmed at the protein level (Figure 3B). We also observed that HS and LPS exerted a synergistic stimulatory effect on the production of some chemokines such as MIP-3α and GROα (Figure 3B). Moreover, and consistent with these observations, evaluation of neutrophil migration by transwell assays showed that supernatants collected from HS-treated neutrophils after 8 h of culture strongly induced the chemotaxis of untreated neutrophils. Of note, the chemotactic response induced by these supernatants was shown to be markedly higher compared with the response induced by supernatants collected from LPS-treated neutrophils (Figure 3C).

### 2.3. High Salt Markedly Inhibits the Transcription of Genes Involved in Mitochondrial Oxidative Phosphorylation While Stimulating the Transcription of FOS, BTK and COX2 Genes: Correlation with Functional Studies

**Figure 4:**
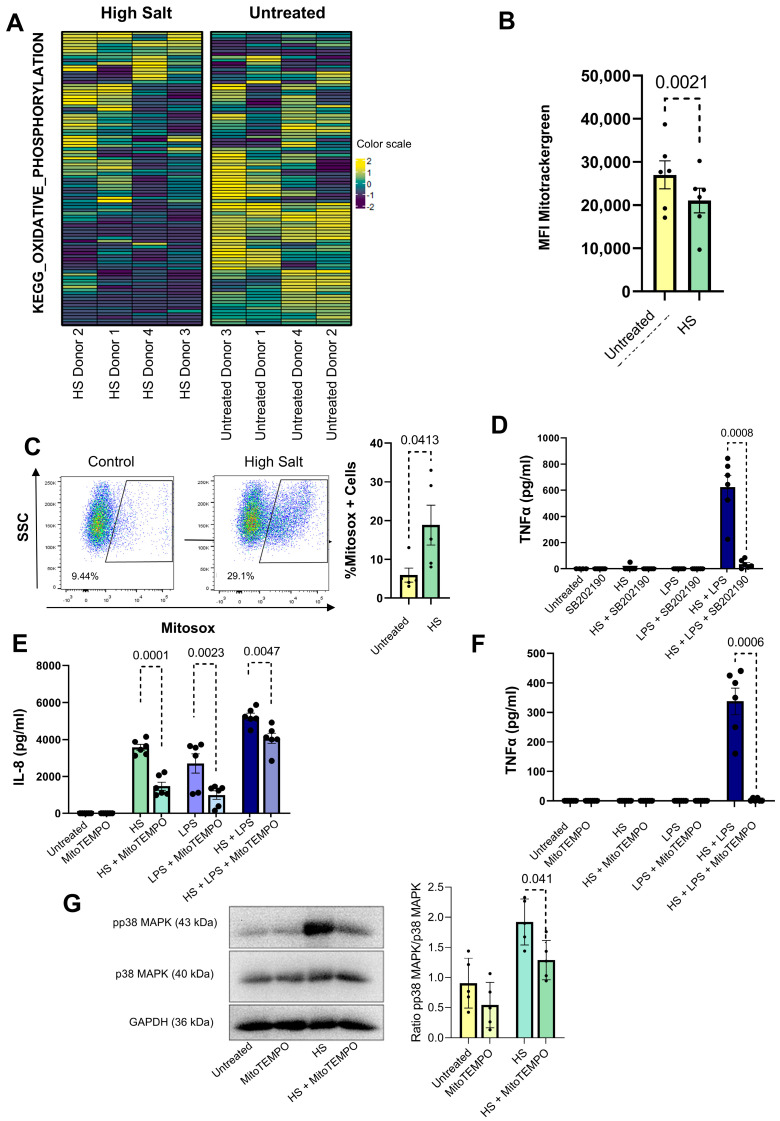
Mitochondrial ROS are involved in the production of IL-8 and TNF-α by high salt-stimulated neutrophils. (**A**) Heatmap showing normalized z-values of genes involved in oxidative phosphorylation (from KEGG oxidative phosphorylation dataset). Columns represent individual samples grouped into two experimental conditions: HS-treated neutrophils and untreated neutrophils. The color scale indicates relative gene expression levels, with hierarchical clustering applied to columns to visualize expression patterns. (**B**) Neutrophils (2 × 10^6^/mL) were cultured for 6 h at 37 °C in culture medium supplemented, or not, with HS. Cells were then washed, resuspended in culture media (without serum) and labeled with MitoTracker Green (100 nM). After 20 min of incubation at 37 °C, mitochondrial mass was measured by flow cytometry. Results are expressed as the mean ± SEM of six donors. (**C**) Neutrophils (2 × 10^6^/mL) were cultured for 6 h at 37 °C in culture medium supplemented, or not, with NaCl 50 mM. Cells were then washed, suspended in PBS, and labeled with MitoSOX (1 μM). After 20 min of incubation at 37 °C, mitochondrial ROS production was evaluated by flow cytometry. A representative experiment and the mean ± SEM of five donors are shown. (**D**) Neutrophils were cultured for 18 h at 37 °C in culture medium supplemented, or not, with NaCl 50 mM and/or LPS (100 ng/mL) in the absence or presence of the p38 MAPK inhibitor SR202190 (10 μM). TNF-α levels were determined by ELISA in culture supernatants. Results are expressed as the mean ± SEM of six experiments. (**E**,**F**) Neutrophils were cultured for 8 h or 18 h at 37 °C in culture medium supplemented, or not, with NaCl 50 mM and/or LPS (100 ng/mL), in the absence or presence of the mitochondrial ROS scavenger MitoTEMPO (10 μM). IL-8 (**E**) and TNF-α (**F**) production was measured by ELISA as described in Materials and Methods. Results are expressed as the mean ± SEM of six experiments. (**G**) Neutrophils (5 × 10^6^/mL) were cultured for 4 h at 37 °C in culture medium supplemented, or not, with NaCl 50 mM, in the absence or presence of MitoTEMPO (10 µM). Western blotting assays were performed as described in Materials and Methods, using anti-p38 and antiphospho-p38 antibodies. Bars represent the mean ± SEM of five experiments.

We found that transcription of mitochondrial genes responsible for oxidative phosphorylation was suppressed in neutrophils exposed to HS (Figure 4A), suggesting impaired mitochondrial function. Consistent with this observation, when analyzing mitochondrial mass by flow cytometry using MitoTracker Green we found a significant reduction in HS-treated neutrophils (Figure 4B). We next analyzed whether the compromise in the mitochondrial compartment was associated with the production of mitochondrial reactive oxygen species (mtROS). By using the mitochondria-targeted superoxide-sensitive fluorescent dye MitoSOX and flow cytometry, we found that exposure to HS stimulated the production of mtROS (Figure 4C). We have previously shown that IL-8 production induced by HS is dependent on the activation of p38-MAPK [52]. Moreover, we reported that HS did not induce the production of TNF-α by neutrophils but markedly stimulated TNF-α production in the presence of LPS [52]. Figure 4D shows that, as observed for IL-8 production induced by HS, TNF-α production induced by HS plus LPS was abolished by the p38-MAPK inhibitor SB202190. Given the large body of evidence indicating that mtROS promote the activation of p38MAPK in myeloid cells [55,56,57], we then analyzed whether the specific mtROS scavenger MitoTempo was able to inhibit IL-8 and TNF-α production by HS-treated neutrophils. As shown in Figure 4E,F, MitoTempo markedly reduced the production of both cytokines, suggesting that modulation of neutrophil function by HS is dependent, at least in part, on changes induced in the mitochondrial compartment resulting in the generation of mtROS and signaling through the p38MAPK pathway. Supporting this view, we found that MitoTEMPO significantly prevented phosphorylation of p38-MAPK (Figure 4G).

**Figure 5:**
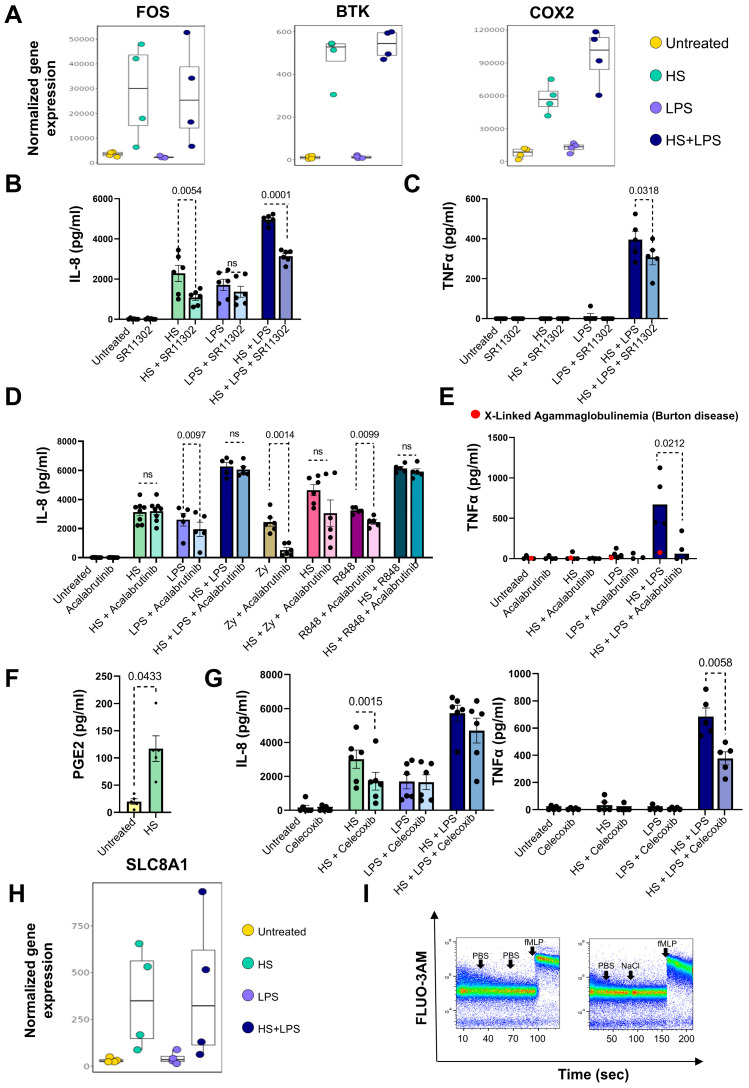
Signaling pathways mediated by FOS, BTK, and COX2 are involved in the acquisition of an inflammatory signature by high salt-exposed neutrophils. (**A**) Normalized gene expression values of *FOS*, *BTK*, and COX-2. (**B**,**C**) Neutrophils at a concentration of 2 × 10^6^/mL or 10 × 10^6^/mL were cultured for 8 h or 18 h at 37 °C in culture medium supplemented, or not, with NaCl 50 mM and/or LPS (100 ng/mL), in the absence or presence of the AP-1 inhibitor SR11302 (10 μM). IL-8 and TNF-α production was measured in supernatants as described in Materials and Methods. Results are expressed as the mean ± SEM of 5–6 experiments. (**D**) Neutrophils (2 × 10^6^/mL) were cultured for 8 h at 37 °C in culture medium supplemented, or not, with NaCl 50 mM and/or LPS (100 ng/mL), Resiquimod (5 μM), or Zymosan (50 μg/mL), in the absence or presence of the BTK inhibitor Acalabrutinib (1 μM). IL-8 levels were determined by ELISA in culture supernatants. Results are expressed as the mean ± SEM of 5–8 experiments. (**E**) Neutrophils (10 × 10^6^/mL) were cultured for 18 h at 37 °C in culture medium supplemented, or not, with NaCl 50 mM and/or LPS (100 ng/mL), in the absence or presence of Acalabrutinib (1 μM). Red dots illustrate a single experiment performed with neutrophils from a patient with X-linked agammaglobulinemia (Bruton disease). TNF-α levels were determined by ELISA in culture supernatants. Results are expressed as the mean of four experiments. (**F**) Neutrophils (10 × 10^6^/mL) were cultured for 18 h at 37 °C in culture medium supplemented, or not, with NaCl 50 mM. PGE2 levels were measured in cell supernatants as described in Material and Methods. Results are expressed as the mean ± SEM of five experiments. (**G**) Neutrophils at a concentration of 2 × 10^6^/mL or 10 × 10^6^/mL were cultured for 8 h or 18 h at 37 °C in culture medium supplemented, or not, with NaCl 50 mM and/or LPS (100 ng/mL), in the absence or presence of celecoxib (10 μM). IL-8 and TNF-α production was measured by ELISA as described in Materials and Methods. Results are expressed as the mean ± SEM of 5–6 experiments. (**H**) Normalized gene expression values of SLC8A1. (**I**) The induction of cytosolic calcium transients was evaluated using neutrophils labeled with fluo-3-AM and flow cytometry. Arrows indicate the time at which PBS, NaCl 50 mM, or fMLP (0.1 µM) were added. A representative experiment (*n* = 3) is shown.

Among the genes that showed an important increase in their transcriptional profile we found *FOS*, *BTK*, and *COX2* (*PTSG2*) (Figure 5A). c-FOS, the protein encoded by the gen *FOS*, is a component of the activator protein-1 (AP-1) transcription factor complex, which plays a crucial role in regulating various cellular processes, including proliferation, differentiation, and apoptosis in response to stress signals [58]. The p38 MAPK/AP-1 pathway has been shown to be activated in neutrophils in response to different stimuli [57,59,60]; however, previous observations have suggested that the production of inflammatory cytokines by neutrophils stimulated by conventional agonists is not under the control of AP-1 [61]. The results in Figure 5B show that IL-8 production by HS-stimulated neutrophils, either in the absence or presence of LPS, was significantly inhibited by the AP-1 inhibitor SR11302. By contrast, IL-8 production by LPS-stimulated neutrophils was slightly but not significantly inhibited by SR11302. Consistent with the inhibition observed for IL-8 production, we found that SR11302 significantly inhibited TNF-α production by neutrophils stimulated by HS plus LPS (Figure 5C). Together, these results suggest the involvement of c-FOS in the stimulation of cytokine production by HS-stimulated neutrophils.

The enzyme BTK plays a critical role in B cell differentiation, expansion, and function [62]. Moreover, it is also involved in the antimicrobial function of macrophages and neutrophils [63,64]. Considering the marked increase in the *BTK* gene transcript observed in HS-treated neutrophils, we analyzed its role by using the BTK inhibitor acalabrutinib. While production of IL-8 stimulated by LPS, Zymosan, or the TLR7/8 agonist resiquimod was significantly inhibited by acalabrutinib, no inhibition was observed in neutrophils stimulated by HS, either in the absence or presence of LPS, zymosan, or resiquimod (RSQ) (Figure 5D). By contrast, the production of TNF-α induced by HS plus LPS was almost completely abolished (Figure 5E). Moreover, the fact that neutrophils isolated from a patient with X-Linked Agammaglobulinemia (Bruton disease) were severely compromised in their ability to produce TNF-α upon stimulation by HS plus LPS (Figure 5E) further suggested a role for BTK in the stimulation of TNF-α production.

Activation of neutrophils by inflammatory agents promotes the expression of COX2 [65,66]. We found that the increased expression of the *COX2* transcripts in HS-treated neutrophils (see Figure 5A) was associated with an enhanced production of PGE2 (Figure 5F). Moreover, we found (Figure 5G) that the specific inhibitor of COX2 Celecoxib did not affect the production of IL-8 induced by LPS but significantly suppressed the production of both IL-8 and TNF-α induced by HS and HS plus LPS, respectively.

Regarding the early events responsible for cellular activation in response to HS, Neubert P and coworkers reported that exposure to HS promoted an inflammatory profile in mouse bone marrow-derived macrophages and that this response is mediated through the activation of the Na^+^/Ca^2+^ exchanger 1 (NCX1), also known as solute carrier family 8 member A1 (SLC8A1), acting as a Na^+^ sensor [67]. This exchanger is able to sense high-Na^+^ environments, triggering Na^+^ influx and concomitant Ca^2+^ efflux [68]. The authors reported that the activation of NCX1 by HS resulted not only in a rapid Na^+^ influx together with a rapid decrease in intracellular Ca^2+^ concentrations but also in the acquisition of an inflammatory signature by macrophages revealed by an increased production of nitric oxide and an enhanced microbicidal activity. Our transcriptomic data show that exposure of neutrophils to HS resulted in an increased expression of the SLC8A1 transcript (Figure 5H). However, contrasting with the observations made in mouse macrophages we found that exposure of neutrophils to HS did not affect either intracellular Ca^2+^ concentration or the induction of Ca^2+^ transients triggered by fMLP (Figure 5I), suggesting that neutrophil activation induced by HS does not involve the participation of NCX1.

**Figure 6:**
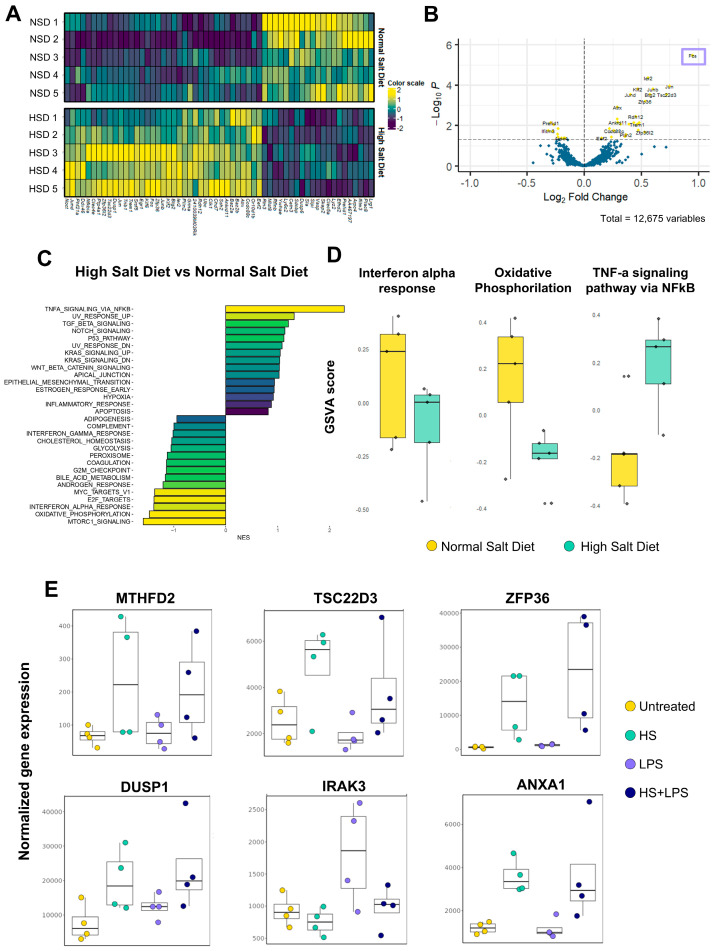
Transcriptome analysis of neutrophils from mice fed a high salt diet. (**A**,**B**) Heatmap and volcano plots show the results of differential gene expression analysis of neutrophils from mice fed a high-sodium diet compared with neutrophils from mice fed a normal salt diet. Differentially expressed genes (DEGs) are defined as adjusted *p*-value < 0.05. (**C**) Gene Set Enrichment Analysis (GSEA) was performed to identify enriched pathways when comparing neutrophils from mice fed a high-sodium diet to those from mice fed a normal salt diet. The fold changes from differential expression analysis were used to assess pathway enrichment using curated HALLMARK signaling pathways. Pathways with positive or negative enrichment scores (ESs) were separately analyzed and the top 15 upregulated and downregulated pathways were visualized in a bar plot. Pathways were sorted based on normalized enrichment scores (NESs), and the color scale represents the adjusted *p*-values for each pathway. (**D**) Gene Set Variation Analysis (GSVA) was used to estimate the enrichment scores of selected hallmark pathways. Box plots display GSVA scores for the Interferon alpha response, Oxidative Phosphorylation, and TNF-α signaling via NF-κB pathways. (**E**) Normalized gene expression values of glucocorticoid target genes in human neutrophils cultured as indicated.

To our knowledge, no previous study has analyzed the transcriptomic changes induced in neutrophils exposed to HS. Interestingly, by re-analyzing published datasets from a study focused on the impact of high-sodium diets on the function of murine neutrophils [69], we found not only clear differences in the transcriptomic signature of neutrophils when compared with mice on a control diet (Figure 6A), but also broad coincidences with a number of our own observations described above. Volcano plots show *FOS* as the most upregulated gene (Figure 6B), while genes involved in mitochondrial oxidative phosphorylation and IFN I signaling were found to be downregulated (Figure 6C,D). Also, in accordance with our observations, an increased expression of genes involved in TNF-α signaling pathways (Figure 6C,D) was found. Finally, while the authors show that the intake of HS diets modulates neutrophil function through a glucocorticoid-dependent mechanism, we found that exposure of neutrophils in vitro to HS stimulated the transcription of a number of glucocorticoid target genes (Figure 6E).

### 2.4. Identification of Novel High Salt-Responsive Genes in the Neutrophil

**Figure 7:**
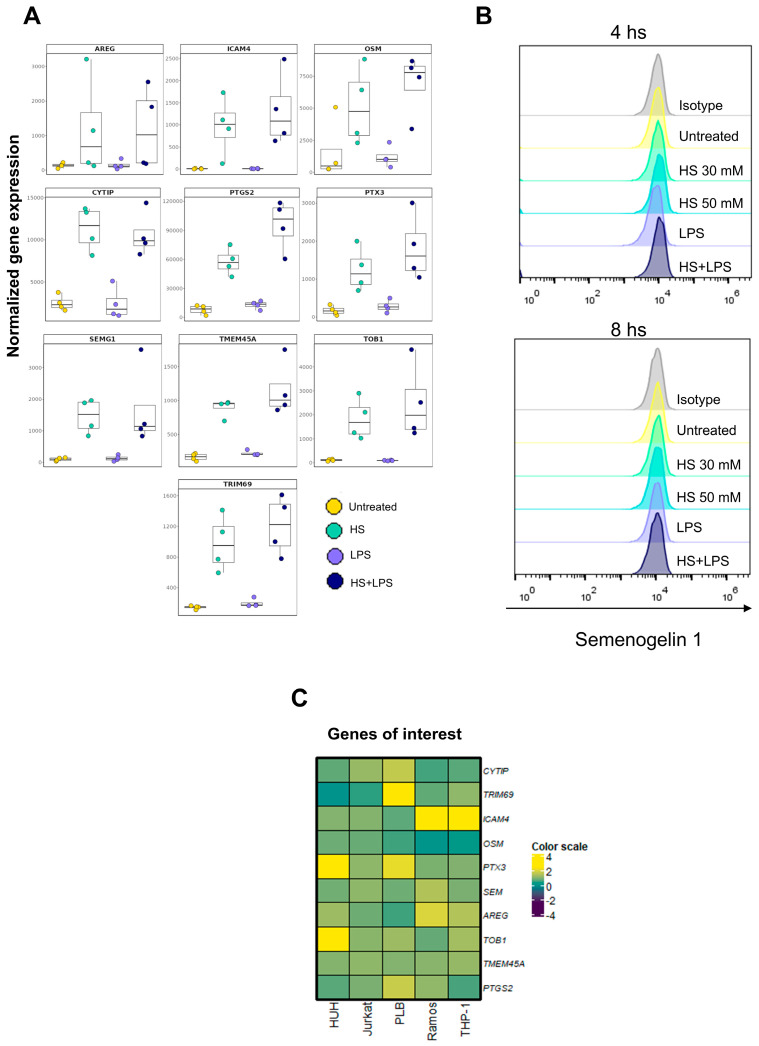
Identification of novel high salt-responsive genes in the neutrophil. (**A**) Normalized expression values of selected genes from neutrophils cultured as indicated. (**B**) Neutrophils at a concentration of 2 × 10^6^/mL were cultured for 4 h or 8 h at 37 °C in culture medium, supplemented or not with NaCl 30 mM or 50 mM and/or LPS (100 ng/mL). Cells were then washed, fixed, permeabilized and incubated with a mouse primary antibody directed against SEMG1, stained with an Alexa Fluor^®^ 488-conjugated anti-mouse secondary antibody, and analyzed by flow cytometry. A representative experiment (*n* = 5) is shown. (**C**) Cell lines were cultured for 4 h at 37 °C in culture medium supplemented or not with 50 mM of NaCl. Cells were then lysed, RNA extracted, and retrotranscribed, and the relative expression levels of *CYTIP*, *TRIM69*, *ICAM4*, *OSM*, *PTX3*, *SEMG1*, *AREG*, *TOB1*, *TMEM45A*, and *PTGS2* were analyzed by qPCR, using *ACTB* as a housekeeping gene. A representative experiment (*n* = 3) is shown.

Considering the profound changes induced by HS on the neutrophil transcriptome and in order to identify novel HS-responsive genes, we selected a group of upregulated genes based on four criteria: (1) negligible expression of the transcript in untreated cells; (2) inability of LPS to increase transcript expression; (3) the absence of prior published evidence in any cell type linking gene expression and HS exposure; and/or (4) the absence of previous reports indicating its expression in neutrophils. This analysis led to the selection of a gene set described in Table 1. Among these were genes coding for semenogelin (*SEMG1*), the major component of human seminal fluid, and ICAM-4, whose expression appears limited to erythroid cells and plays a relevant role in the differentiation of healthy erythroblasts [70]. Transcript expression in neutrophils exposed to HS and/or LPS is shown in Figure 7A. We found that HS markedly stimulated the transcription of these genes, which are involved in a wide diversity of functions including stimulation of cell proliferation, cellular adhesion, promotion of inflammatory reactions, activation of the complement system, and antibacterial and antiviral activity, among others. We next sought to validate these observations at the protein level by assessing SEMG1 expression in HS-treated neutrophils. However, no detectable protein expression was observed (Figure 7B), suggesting that transcript abundance does not necessarily predict protein levels.

We then asked ourselves whether HS was also able to imprint a common transcriptional signature in different cell types. To this aim, we used the B cell line Ramos, the T cell line Jurkat, the myeloid leukemia cell line PLB-985, the monocytic leukemia cell line THP-1, and the hepatocellular carcinoma cell line HUH-7. These cell lines were exposed to HS for 4 h, and the expression of gene transcripts were evaluated by qPCR. As shown in Figure 7C, the response induced by HS markedly varied across cell lines, displaying a heterogeneous expression pattern distinct from that observed in neutrophils. This observation suggests that the impact of HS on the cell transcriptome is highly dependent on the cell type analyzed.

## 3. Discussion

We here report that exposure to HS resulted in a broad transcriptomic response in human neutrophils. Of note, these changes involved the upregulation and downregulation of 2499 and 2098 transcripts, respectively, these changes being markedly higher than those induced by LPS. Over-representation analysis showed that genes involved in MAPK signaling, a pathway involved in the response to stressful conditions and conventional agonists resulting in the production of a number of cytokines [82], represented the most significantly enriched pathway in HS-exposed neutrophils. Our observations suggest that the production of both IL-8 and TNF-α induced by HS involves the activation of p38-MAPK. The mechanisms leading to the activation of this pathway remain to be defined; however, they appear to involve the participation of mtROS. In fact, and in agreement with a previous report [83], we found that exposure to HS triggers the production of mtROS by neutrophils. Also consistent with this observation, it has been previously reported that HS transiently inhibits mitochondrial function in monocytes and macrophages, resulting in the production of mtROS and the subsequent activation of the inflammatory and bactericidal activity of mononuclear phagocytes [50,84]. A large body of evidence not only supports a role of mtROS in the activation of mononuclear phagocytes [85,86,87,88] and neutrophils [89,90,91], but also that mtROS are able to induce the activation of p38MAPK in myeloid cells [55,56,57]. Our observations suggest a role for mtROS in the activation of neutrophils exposed to HS. In fact, we found not only that HS triggered the production of mtROS but also that the scavenger of mtROS MitoTEMPO significantly inhibited both the phosphorylation of p38MAPK and the production of IL-8 and TNF-α.

Analysis of chemokine genes, on the other hand, revealed a strong upregulation of chemokines responsible for the recruitment of neutrophils at inflammatory foci, including not only *CXCL8* but also *CXCL6*, *CXCL3*, *CXCL2*, and *CXCL1*. Interestingly, for all these chemokine genes we found that HS induced a stronger response compared with LPS, and consistent with this observation, when analyzing the biological activity of supernatants from HS-treated neutrophils we observed a chemotactic response markedly higher compared to the response induced by supernatants from LPS-stimulated neutrophils. Contrasting with the observations made in chemokine genes involved in the recruitment of neutrophils we found that *CCL2*, which encodes the most important chemokine responsible for the recruitment of monocyte (MCP-1), was downregulated at both transcript and protein levels.

We also found that exposure of neutrophils to HS increased the expression of the *COX-2* transcript and the production of PGE2, while the inhibition of COX-2 by Celecoxib significantly inhibited the production of IL-8 and TNF-α promoted by HS. Consistent with this observation, it has been previously reported that inhibition of COX-2 in neutrophils suppressed the production of inflammatory cytokines by neutrophils stimulated by either the chemotactic peptides fMLP or opsonized zymosan [92]. Interestingly, Zhang M-Z and coworkers reported in a mouse model that a high-sodium diet induced the expression of COX-2 in kidney and skin macrophages, promoting homeostasis in response to chronically increased dietary salt through a mechanism dependent, at least in part, on an increased lymphangiogenesis and a lower retention of sodium excess [93]. The mechanisms through which HS induced the expression of COX-2 remain to be clarified. We hypothesized that it might involve the oxidative stress mediated by mtROS. In fact, mtROS have been shown to induce the expression of COX-2 in different cell types [94,95,96].

Our transcriptomic analysis used neutrophils treated with HS for 4 h, while functional experiments were performed after 6, 8, or 18 h of treatment. While it is reasonable to assume that the functional observations made at 6 and 8 h of culture largely reflect the transcriptional changes analyzed at 4 h, the functional outcomes observed at 18 h may involve later transcriptional events that were not assessed in this study. Transcriptomic analyses conducted at later time points, such as 12 h of culture, may provide a more accurate correlation with these late responses.

To the best of our knowledge, no previous work has analyzed the transcriptomic response of human neutrophils exposed to HS. In studies aiming to analyze the impact of a HS diet on murine neutrophils, Jobin K et al. showed that it compromises neutrophil antibacterial response by inducing hormonal perturbation, i.e., through a glucocorticoid-dependent manner [69]. By analyzing the transcriptomic data generated by the authors in murine neutrophils we found a number of similarities with our own data, i.e., a profound downregulation of the expression of genes involved in both oxidative phosphorylation and interferon alpha response, together with a marked increase in *FOS* expression. By contrast, no upregulation in the expression of chemokine genes responsible for neutrophil recruitment was observed. Interestingly, while the authors showed that alterations of neutrophil function in vivo are mediated through a glucocorticoid-dependent mechanism, we found that exposure of human neutrophils to HS in vitro resulted in the upregulation of known glucocorticoid target genes [97] such as *TSC22D3*, *MTHFD2*, *ZFP36*, *ANXA1*, and *DUSP1*. This would suggest the existence of some common signaling pathways induced in neutrophils by glucocorticoids and HS. On the other hand, we compared our transcriptional data with those previously reported in monocytes [54] and macrophages [51] exposed in vitro to HS. Only a few transcripts were commonly modulated when neutrophils (our own data) were compared with monocytes and macrophages, suggesting the acquisition of different transcriptomic signatures within the myeloid compartment in response to HS. However, this suggestion should be interpreted with caution, because our transcriptomic data in neutrophils were obtained at 4 h of culture, while observations made in monocytes and macrophages were obtained after 24–72 h of culture.

Our study has a number of limitations. First, transcriptomic analyses were performed using samples from only four healthy donors. Although the results were consistently reproduced across these samples, the generalizability and statistical robustness of our findings would be strengthened by including a larger cohort of donors. Second, although the identification of novel responsive genes such as *SEMG1*, *ICAM-4*, *TRIM69*, *TOB1*, and *TMEM45A* represents an intriguing finding, these results have yet to be validated at the protein level. In fact, while we observed a good correlation between mRNA and protein levels for several cytokines and chemokines, including TNF-α, IL-8, CCL20, and CXCL1, no SEMG1 expression was detected by flow cytometry, despite the marked increase in *SEMG1* transcript levels induced by HS. Consistent with previous reports [98,99], this finding suggests that mRNA abundance does not necessarily predict protein expression in neutrophils.

In summary, our results not only shed light on the mechanisms through which HS promotes the activation of neutrophils but also reinforces the notion that neutrophils are highly plastic cells and suggest that they could modulate biological responses beyond their role in the immune response.

## 4. Materials and Methods

### 4.1. Reagents

Sodium chloride (S5886), dihydrorhodamine-123 (D1054), LPS from *E. coli* (L2630), fMLP (F3506), Zymosan from *S. cerevisiae* (Z4250), and Resiquimod (SML0196) were obtained from Sigma-Aldrich (Buenos Aires, Argentina). SB202190 (10010399), SR11302 (16338), Acalabrutinib (19899), and Celecoxib (10008672) were obtained from Cayman Chemical Company (Ann Arbor, MI, USA). Human Proinflammatory Chemokine Panel (740985) was from Biolegend (San Diego, CA, USA). SYBR Green PCR Master Mix was from Applied Biosystems (Fister City, CA, USA). MitoTracker™ Green FM (M7514) and MitoSox Red (M36007) were from Thermo Fisher (Buenos Aires, Argentina). Human IL-8 (130-122-354) was from Miltenyi Biotec (Auburn, CA, USA), and NiCl_2_ was from Biopack (Buenos Aires, Argentina). The Cisbio HTRF kit (62P2APEG) was obtained from Revvity Health Sciences (San Diego, CA, USA).

### 4.2. Neutrophil Isolation and Culture

Heparinized blood samples were obtained from healthy donors. All of them signed the informed consent. Neutrophils were purified by centrifugation of blood samples on Ficoll-Paque (GE Healthcare, Chicago, IL, USA) and dextran (Sigma-Aldrich) sedimentation. As a first step, blood samples (20 mL) were diluted (1:1 *v*/*v*) with saline (0.9% *w*/*v* NaCl) at room temperature to a final volume of 40 mL. Diluted blood samples were centrifuged on Ficoll-Paque (density 1.077 g/mL) at 400× *g* for 40 min at room temperature in a sterile 50 mL conical plastic tube. Then, the fractions containing saline and plasma and the layer containing mononuclear cells were discarded. The PMN-erythrocyte pellet was resuspended in 25 mL of saline and mixed with 25 mL of 3% *w*/*v* of Dextran-500. The tubes were placed in a vertical position for 30 min at room temperature, and the leukocyte-rich upper layer was then harvested. Contaminating erythrocytes were eliminated by hypotonic lysis, restoring isotonicity after 30 s. Cells were centrifuged at 300× *g* for 10 min at 4 °C, and cell pellets were washed twice with saline. Cell suspensions were labeled with PE-anti-CD14 antibodies and analyzed by flow cytometry (BD FACSCanto cytometer/BD FACSDiva software, BD Biosciences, San Jose, CA, USA) to determine the percentage of contaminant monocytes. In all cases, monocyte contamination was <0.4%. Finally, neutrophils (purity greater than 98%) were suspended in RPMI-1640 medium (Gibco Invitrogen, Carlsbad, CA, USA) supplemented with heat-inactivated fetal calf serum (FCS, 10%), streptomycin (50 μg/mL), and penicillin (50 U/mL), (Gibco Invitrogen). Unless otherwise stated, all the experiments were performed in this culture medium.

### 4.3. RNAseq Library Preparation

Neutrophils (2 × 10^6^/mL, 5 mL) were cultured for 4 h at 37 °C in 5% CO_2_ in culture medium supplemented, or not, with 50 mM NaCl, in the absence or presence of LPS (100 ng/mL). Then, cells were centrifuged and pellets (10 × 10^6^ neutrophils) from each experimental condition were lysed for RNA extraction. Total RNA was isolated using a QIAGEN RNeasy Mini Kit according to the manufacturer’s instructions. Briefly, cells were lysed in Buffer RLT supplemented with 1% β-mercaptoethanol. An equal volume of ethanol 70% was added to the lysate and transferred to the RNeasy spin column. After centrifugation, the flow through was discarded. This was followed by on-column DNase I treatment. Then, 80 μL of DNase I mix (70 μL Buffer RDD with 10 μL DNase I) was added directly to the RNA spin column, incubated for 10 min at room temperature, and then washed. Finally, total RNA was eluted in 50 μL RNase-free water and stored at −80 °C. RNA-seq libraries were prepared using a KAPA mRNA Hyperprep kit (Roche, Buenos Aires, Argentina) according to manufacturer’s instruction. First, poly A RNA was isolated from 300 ng total RNA using oligo-dT magnetic beads. Purified RNA was then fragmented at 85 °C for 6 min, targeting fragments in the range of 250–300 bp. Fragmented RNA was reverse transcribed with an incubation of 25 °C/10 min, 42 °C/15 min, and an inactivation step at 70 °C/15 min. This was followed by second strand synthesis and A-tailing at 16 °C/30 min and 62 °C/10 min. A-tailed, double stranded cDNA fragments were ligated with Illumina unique dual indexed adapters (Illumina, San Diego, CA, USA). Adapter-ligated DNA was purified using Ampure XP beads. This was followed by 10 cycles of PCR amplification. The final library was cleaned up using AMpure XP beads. Quantification of libraries was performed using real-time qPCR (Thermo Fisher, Buenos Aires, Argentina). Sequencing was performed on the Illumina NovaSeq platform generating paired end reads of 150 bp.

### 4.4. RNAseq Data Analysis

FastQ files were pseudoaligned to the human reference Ensembl transcriptome GRCh38 using Kallisto v.0.48.0,108 and a raw count matrix was generated using tximport.118. Analysis was performed using Rstudio 2024.04.2+764, running R DESeq2 R package version 4.4.0 was used for the analysis of differentially expressed genes [100], after filtering lowly expressed genes (<10 counts per row). For the analysis of differential expression, donor interindividual variability was considered as a covariate and accounted for in the statistical comparisons. Genes were considered differentially expressed among comparisons when adjusted *p*-value < 0.05 (Benjamini and Hochberg method), using a Fold Change cutoff of 1.5 (Log2 0.58). Enhanced volcano R package was employed to produce volcano plots. Heatmaps were generated using the heatmap.1, heatmap.2, and pheatmap packages. The rest of the plots were made using ggplot2. Gene Set Enrichment Analysis was performed using the fgsea package version 3.21 [101]. Normalized gene expression data can be interactively accessed at https://ignacio21.shinyapps.io/NeutrophilsUBA/ (accessed on 3 October 2024). In addition to the main RNA-seq dataset, we analyzed three independent datasets from human monocyte-derived macrophages [51] (GSE68482), human monocytes [54] (https://figshare.com/s/d810937dc537eeb361a5?file=23086487, accessed on 25 October 2024), and neutrophils from mice fed a high-sodium diet [69] (PRJEB28204). All datasets were processed using a similar pipeline, with no direct comparisons made between them.

### 4.5. Neutrophil Chemotaxis Assays

Neutrophils (5 × 10^6^/mL) were incubated at 37 °C and 5% CO_2_ for 18 h in medium supplemented, or not, with NaCl (50 mM) or LPS (100 ng/mL), and cell supernatants were then collected. Supernatants from HS-stimulated neutrophils were adjusted to iso-osmolarity by adding distilled water. Migration assays were performed as described [102], using untreated neutrophils and a transwell chamber featuring uncoated polyester membrane with 5-µm pores (Corning, Corning, NY, USA). Untreated neutrophils (1.5 × 10^5^/100 µL) suspended in culture medium supplemented with 10% fetal calf serum were added to the upper compartment of the chamber. 100 µL of culture medium (control), culture medium supplemented with 50 mM NaCl, or neutrophil supernatants obtained as described above, were added to the lower compartment of the chamber. As a positive control of chemotaxis, we added IL-8 (10 ng/mL) to the lower compartment of the chamber. A positive control of chemokinesis was performed by adding IL-8 to both the upper and the lower compartment of the transwell chamber. As additional controls we included HS (50 mM NaCl) or IL-8 (10 ng/mL), added in the lower chamber. After incubation for 1 h at 37 °C and 5% CO_2_, neutrophils in the upper and lower chamber were harvested and quantified by flow cytometry. Results are expressed as the percentage of neutrophils that migrated from the upper chamber to the lower chamber.

### 4.6. Measurement of Cytokines

Neutrophils (2 × 10^6^/mL or 10 × 10^6^/mL) were cultured for 8 h or 18 h at 37 °C in 5% CO_2_ in culture medium supplemented or not with 50 mM of NaCl, in the absence or presence of LPS (100 ng/mL), zymosan (50 μg/mL), or Resiquimod (10 µM). IL-8 and TNF-α production was measured by ELISA according to the manufacturer’s recommendations (BD Biosciences, San Jose, CA, USA). IL-8 was measured in supernatants from neutrophils cultured for 8 h at a concentration of 2 × 10^6^ cells/mL while TNF-α production was measured in supernatants from neutrophils cultured for 18 h at a concentration of 10 × 10^6^ cells/mL. A panel of proinflammatory chemokines was also measured in neutrophil supernatants using LEGENDplex Human Proinflammatory Chemokine Panel 1 (Biolegend, San Diego, CA, USA)).

### 4.7. Analysis of PGE2 Concentrations by Homogeneous Time-Resolved Fluorescence Assay (HTRF)

PGE2 levels were measured in culture supernatants using the HTRF kit (Cisbio, Bedford, MA, USA) following the manufacturer’s protocol. A standard curve (6.85–5000 pg/mL) was prepared by serial dilutions (1:3). Samples were plated in a 96-well plate. Reagents were then added and plates were incubated overnight at 4 °C. Fluorescence was analyzed by using a Flexstation 3 microplate reader. Data were analyzed by calculating the delta F (%).

### 4.8. Measurement of Mitochondrial Mass and Mitochondrial ROS

Neutrophils (2 × 10^6^/mL) were cultured at 37 °C and 5% CO_2_ for 8 h in culture medium supplemented or not with 50 mM NaCl. Then, neutrophils were washed, resuspended in culture medium, and stained with MitoSOX (5 μM) or MitoTracker Green (100 nM). Mitochondrial mass and mitochondrial ROS were then analyzed by flow cytometry.

### 4.9. Phosphorylation of p38 MAPK

Neutrophils were lysed in radioimmunoprecipitation assay (RIPA) buffer supplemented with phosphatase inhibitors (PhosSTOP, Roche, Buenos Aires, Argentina) and a protease inhibitor cocktail (Sigma-Aldrich). Total protein concentration was measured using the Pierce BCA Protein Assay Kit (Thermo Fisher, Buenos Aires, Argentina). Equal amounts of protein (20 µg) were denatured by boiling and resolved on 10% SDS–polyacrylamide gels. Phosphorylation of p38 MAPK was assessed by immunoblotting with antibodies specific for the phosphorylated and total forms of p38 (Cell Signaling Technology, Danvers, MA, USA), while GAPDH was used as a loading control. Membranes were blocked for 1 h at room temperature in 5% non-fat milk prepared in Tris-buffered saline containing 0.05% Tween-20 (TBST) and then incubated overnight at 4 °C with primary antibodies diluted 1:1000. Following three washes (5 min each) with TBST, membranes were incubated with horseradish peroxidase (HRP)-conjugated secondary antibodies (Jackson ImmunoResearch Laboratories, West Grove, PA, USA) at a dilution of 1:10,000. Protein bands were visualized using the SuperSignal™ West Pico PLUS Chemiluminescent Substrate (Thermo Fisher, Buenos Aires, Argentina), and signals were captured using the BioSpectrum-815 Imaging System (Upland, CA, USA).

### 4.10. Analysis of Calcium Transients

Changes in intracellular concentrations of free calcium ([Ca^2+^]i) were analyzed using fluo-3-AM (BD Pharmingen, Waltham, MA, USA). Neutrophils, at a concentration of 2.5 × 10^6^ cells/mL, were treated with 4 µM fluo-3-AM for 30 min at 30 °C. Then, cells were washed, resuspended at 2.5 × 10^6^ cells/mL in culture medium, incubated at 37 °C, and analyzed by flow cytometry.

### 4.11. Quantitative Real-Time PCR (qPCR)

Cells were incubated for 4 h at 37 °C in 5% CO_2_ in medium supplemented or not with NaCl 50 mM. Cells were then lysed and RNA was extracted using a PureLink RNA Mini Kit (ThermoFisher, Buenos Aires, Argentina). Then, 100–200 ng of RNA was retrotranscribed into cDNA using Promega M-MLV reverse transcriptase in the presence of dNTPs and random primers. qPCR was performed using SYBR Green PCR Master Mix (Applied Biosystems, Foste City, CA, USA) and specific forward and reverse primers (250 nM). The primer sequences used were as follows:
Human PTX3_FAGGAAGGGCTCACATCCTTGHuman PTX3_RTGACCTGTGGCCATCTCAACHuman SEMG1_FTGGATCTCATGGGGGATTGGAHuman SEMG1_RAATGGGTTTCGGTCGTTGTTHuman AREG_FAGCACCTGGAAGCAGTAACAHuman AREG_RCGTATTGTCTTCTAAGCTGGACTGHuman TOB1_FGAGCCACTAACGGCGATCTHuman TOB1_RGATCTTGTTCGGGCTGCTTCHuman TMEM45A_FGAGCTGGTTCTTTCAGATTGGAHuman TMEM45A_RCTGAGGAGCAGAGCCTCTTAHuman PTGS2_FCCCTTCTGCCTGACACCTTTHuman PTGS2_RTTCTGTACTGCGGGTGGAACHuman TRIM69_FTCCATTTCTTCACGGAGGAGCHuman TRIM69_RCCATGGACACATGTTGCTGCHuman CYTIP_FGGCCACTCAACCATGTGCTAHuman CYTIP_RTGTGTTGCAGGAGCCTTTGTHuman RBBP6_FCGTAGGCGCTCATTTTCCAGHuman RBBP6_RTGCCTCTTCTGGGGTATGGAHuman IRF4_FTTTATGCTTGTGCCCCACCTHuman IRF4_RTCGGCAGACCTTATGCTTGGHuman ICAM4_FTGATTTTGGAGCCTCCGGTCHuman ICAM4_RTCCAGCAGCAAACTCGTAGGHuman OSM_FATGGGGGTACTGCTCACACAHuman OSM_RATAGGGGTCCAGGAGTCTGCHuman OAS1_FACAGGAAACTTGGGTGGTGGHuman OAS1_RGTGCAGGTCCAGTCCTCTTCHuman IFIT1_FAAACAGATTGCCTCCTCCCTHuman IFIT1_RTTCCCACACTGTATTTGGTGTC

### 4.12. Analysis of SEMG1 by Intracellular Staining and Flow Cytometry

Neutrophils (2 × 10^6^ cells/mL) were cultured at 37 °C in a humidified 5% CO_2_ atmosphere for 4 or 8 h, in culture medium supplemented or not with 30 mM or 50 mM NaCl, in the presence or absence of LPS (100 ng/mL). Then, cells were washed and subsequently fixed and permeabilized using the BD Cytofix/Cytoperm™ Kit (BD Biosciences, San Jose, CA, USA), according to the manufacturer’s instructions. Permeabilized cells were incubated with a primary antibody directed against SEMG1 (Santa Cruz, Dallas, TX, USA), washed with a Perm/Wash buffer, and then stained with an Alexa Fluor^®^ 488-conjugated anti-mouse secondary antibody (Jackson ImmunoResearch Laboratories, West Grove, PA, USA). Finally, samples were analyzed using flow cytometry to assess intracellular SEMG1 expression.

### 4.13. Statistical Analysis

When two groups were present, normally distributed data were analyzed by a two-sided t test and skewed data were analyzed by a Wilcoxon test. For three or more groups, one-way ANOVA or Kruskal–Wallis tests were employed. Normality was analyzed using the Shapiro–Wilk test. Data analysis was carried out using GraphPad Prism software version 8. Differences were considered to be statistically significant at *p*-values < 0.05.

## Figures and Tables

**Table 1:** Novel high salt responsive genes in the neutrophils.

Gene	Abbreviation	Main Functions
Amphiregulin	*AREG*	Ligand of the epidermal growth factor receptor (EGFR). Promotes cell survival and proliferation in different cell types [71].
Intercellular adhesion molecule 4	*ICAM4*	It binds to VCAM-1 expressed by macrophages promoting erythropoiesis [72].
Oncostatin M	*OSM*	It promotes inflammatory processes and tumor growth [73].
Prostaglandin-endoperoxide synthase 2	*PTGS2*	Key enzyme in the production of prostaglandins [74].
Pentraxin 3	*PTX3*	Soluble pattern recognition receptor that promotes the course of inflammatory processes [75].
Retinoblastoma-binding protein 6	*RBBP6*	It plays a key role in cell proliferation, differentiation, and nucleic acid metabolism [76].
Semenogelin 1	*SEMG1*	Major semen protein that is able to promote semen coagulation, modulate sperm motility, and mediate antibacterial activity [77].
Transmembrane protein 45A	*TMEM45A*	Its physiological function has not yet been determined. It could play a role in tumor progression and resistance to antineoplastic treatments [78].
Transducer of ERBB2.1	*TOB1*	A tumor-suppressor protein that inhibits the proliferation of different cell types [79].
Tripartite motif containing 69	*TRIM69*	Mediates an antiviral action and modulates cell proliferation [80].
Cytohesin 1 interacting protein	*CYTIP*	Regulates leukocyte adhesion [81].

## Data Availability

The data presented in this study are openly available in GEO under the accession number GSE297042, using the token ipgxegawbvupnut.

## Supplementary Materials

The following supporting information can be downloaded at https://www.mdpi.com/article/10.3390/ijms27021083/s1.
