# Supplementary material for: Transcriptomic Analysis Reveals Novel Mechanisms Underlying Neutrophil Activation Induced by High Salt
Source: Int J Mol Sci. 2026 Jan 21;27(2):1083. doi: 10.3390/ijms27021083 (PMC12841887; doi:10.3390/ijms27021083)
Supplement: Supplementary file 1 [file ijms-27-01083-s001.zip › Figure S1.pdf]

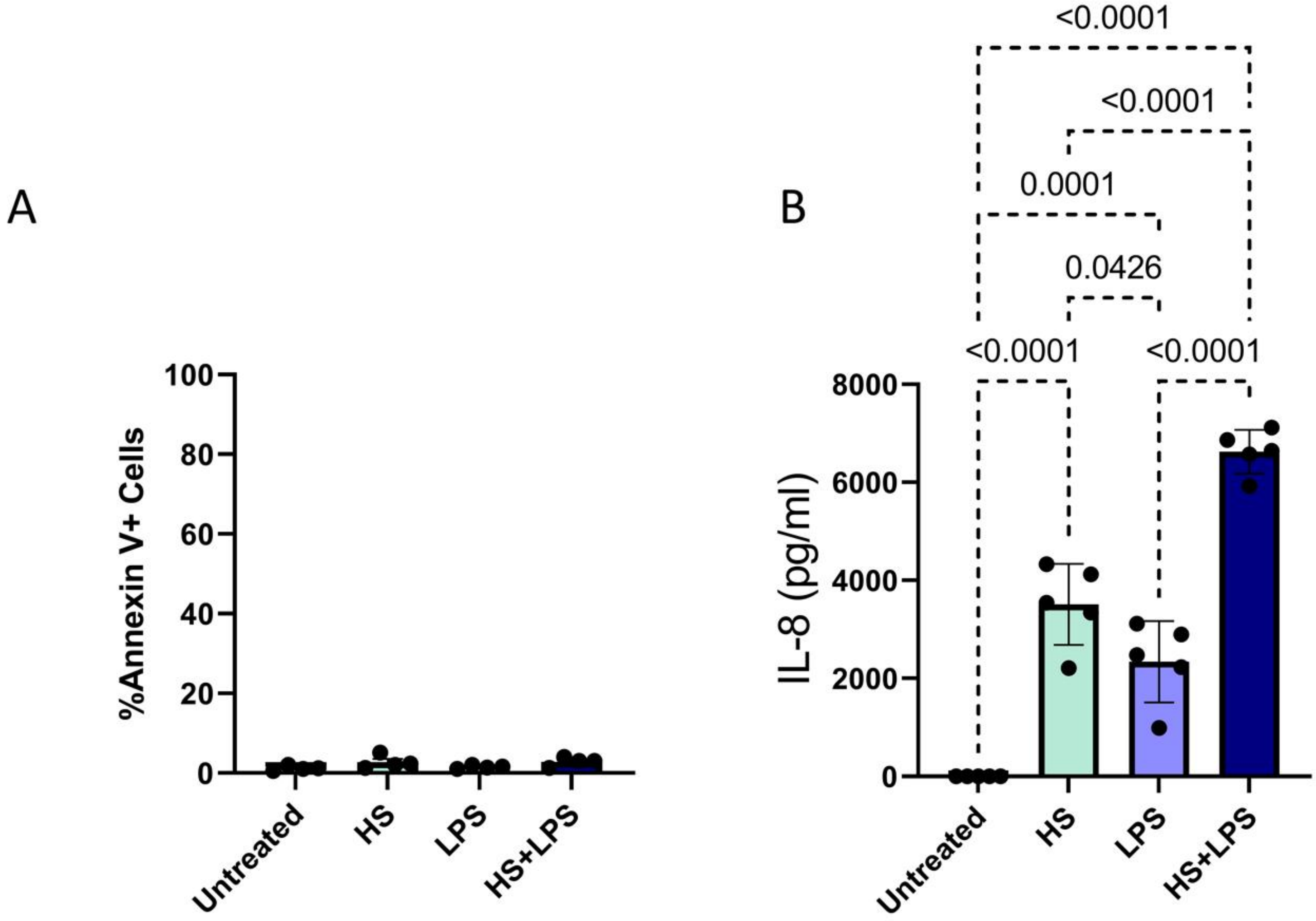


**Figure S1.** (**A**) Neutrophils (2 × 10[6]/mL) were incubated at 37 °C for 4 h in medium supplemented, or not, with NaCl (50 mM) with or without LPS (100 ng/mL). Then cells were labeled with annexin-V FITC and propidium iodide, and apoptosis was evaluated by flow cytometry. The mean ± SE from 4 experiments is shown. (**B**) Neutrophils (2 × 10[6]/mL) were cultured for 8 h at 37 °C in culture medium supplemented, or not, with NaCl 50 mM and/or LPS (100 ng/mL). IL-8 levels were determined by ELISA in culture supernatants. The mean ± SE from 14 experiments is shown.
