# Supplementary material for: Transcriptomic Analysis Reveals Novel Mechanisms Underlying Neutrophil Activation Induced by High Salt
Source: Int J Mol Sci. 2026 Jan 21;27(2):1083. doi: 10.3390/ijms27021083 (PMC12841887; doi:10.3390/ijms27021083)
Supplement: Supplementary file 1 [file ijms-27-01083-s001.zip › Table S1.pdf]

Table S1. Characteristics of healthy donors used for transcriptomic studies.

Donor	Age	Gender	Etnicity
1	25	Male	Caucasian
2	34	Male	Caucasian
3	47	Female	Caucasian
4	31	Female	Caucasian
